# Persistent organ dysfunction plus death: a novel, composite outcome measure for critical care trials

**DOI:** 10.1186/cc10110

**Published:** 2011-03-18

**Authors:** Daren K Heyland, John Muscedere, John Drover, Xuran Jiang, Andrew G Day

**Affiliations:** 1 Department of Medicine, Queen's University, 76 Stuart Street, Kingston, ON K7L 2V7, Canada; 2 Department of Surgery, Queen's University, 76 Stuart Street, Kingston, ON K7L 2V7, Canada; 3 Clinical Evaluation Research Unit, Kingston General Hospital, Kingston, ON K7L 2V7, Canada

## Abstract

**Introduction:**

Due to resource limitations, few critical care interventions have been rigorously evaluated with adequately powered randomized clinical trials (RCTs). There is a need to improve the efficiency of RCTs in critical care so that more definitive high quality RCTs can be completed with the available resources. The objective of this study was to validate and demonstrate the utility of a novel composite outcome measure, persistent organ dysfunction (POD) plus death, for clinical trials of critically ill patients.

**Methods:**

We performed a secondary analysis of a dataset from a prospective randomized trial involving 38 intensive care units (ICUs) in Canada, Europe, and the United States. We define POD as the persistence of organ dysfunction requiring supportive technologies during the convalescent phase of critical illness and it is present when a patient has an ongoing requirement for vasopressors, dialysis, or mechanical ventilation at the outcome assessments time points. In 600 patients enrolled in a randomized trial of nutrition therapy and followed prospectively for six months, we evaluated the prevalence of POD and its association with outcome.

**Results:**

At 28 days, 2.3% of patients had circulatory failure, 13.7% had renal failure, 8.7% had respiratory failure, and 27.2% had died, for an overall prevalence of POD + death = 46.0%. Of survivors at Day 28, those with POD, compared to those without POD, had a higher mortality rate in the six-month follow-up period, had longer ICU and hospital stays, and a reduced quality of life at three months. Given these rates of POD + death and using a two-sided Chi-squared test at alpha = 0.05, we would require 616 patients per arm to detect a 25% relative risk reduction (RRR) in mortality, but only 286 per arm to detect the same RRR in POD + mortality.

**Conclusions:**

POD + death may be a valid composite outcome measure and compared to mortality endpoints, may reduce the sample size requirements of clinical trials of critically ill patients. Further validation in larger clinical trials is required.

## Introduction

In the critical care setting, randomized controlled trials (RCTs) focusing on clinically important endpoints have become the preferred source of evidence on which to base clinical recommendations. However, due to resource limitations, few critical care interventions have been rigorously evaluated with adequately powered RCTs. There is a need to improve the efficiency of RCTs in critical care so that more definitive high quality RCTs can be completed with the available resources.

To judge the efficacy of new interventions or therapies, clinicians and researchers consider the treatment effect of the new intervention on clinically important primary outcome(s). Historically, 28-day mortality has been used as the primary endpoint for large scale trials of critical care interventions. In the last decade, there has been increasing awareness of other endpoints, such as organ failure, infectious complications, and quality of life and a movement beyond the 28-day window to longer-term outcomes, such as hospital survival or six-month quality of life [1,2]. The sample size required to demonstrate whether an intervention is effective or not is determined by the choice and frequency of the primary outcome. Composite endpoints, that combine several clinically related endpoints into an additive outcome measure, are commonly used in other disciplines as a way of enhancing the statistical efficiency and, thereby, reducing the costs of clinical trials. Typically, mortality is combined with other non-fatal endpoints to capture an overall assessment and achieve a higher event rate, thus, reducing the sample size required to show a treatment effect. For example, in cardiology trials, non-fatal myocardial infarctions, hospitalizations, episodes of revascularizations, and stroke have been combined with death in the form of a composite endpoint. This approach also avoids multiple tests of significance and its impact on type 1 errors when endpoints are tested individually.

The purpose of this paper is to propose a novel composite endpoint for critical care trials, the Persistent Organ Dysfunction (POD) combined with death. POD builds on our understanding of multiple organ dysfunction, which is central to the pathogenesis of death and disability in critically ill patients. Several scoring systems have been developed to quantify the degree of organ dysfunction during the initial phase of critical illness [3-5]. Some preliminary work validating Sequential Organ Failure Assessment (SOFA) score, or changes thereof, as an early outcome measure has been published. Vincent and colleagues showed that resolution of SOFA scores over the first seven days is associated with lower 28-day mortality while the development of new organ failures during the first seven days is associated with increased 28-day mortality [6]. However, no organ failure scoring system has been validated as an outcome measure at 28 days or more. Furthermore, all current scoring systems are limited by the lack of biochemical test results or detailed clinical variables in the latter stages of illness, particularly when patients are discharged from ICU. At 28 days, up to 52% of variables necessary to calculate SOFA scores or organ-failure free days are missing [6].

We define POD as the persistence of organ dysfunction requiring life-sustaining technologies and it is present when a patient has an ongoing requirement for vasopressors, dialysis, or mechanical ventilation at the outcome assessments time points. Using a database from a recent randomized trial, we evaluate the prevalence of POD and the validity of combining POD + death. To validate POD, we determine whether POD is associated with poor health outcomes at three and six months. To demonstrate the statistical utility of POD, we compare sample size calculations based on POD to similar calculations using other conventional outcomes.

## Materials and methods

This study is a secondary analysis of a cohort of patients enrolled in a prospective randomized trial to evaluate the efficacy of supplemental glutamine and antioxidant strategies in critically ill patients (REducing Deaths due to OXidative Stress: The REDOXS study, registered at clinicaltrials.gov NCT00133978). The details of this trial have been published elsewhere [7]. In brief, we enrolled mechanically-ventilated adult patients (≥18 years old) admitted to ICU with two or more of the following organ failures related to their acute illness: 1. A PaO2/FiO2 ratio of ≤300; 2. Clinical evidence of hypoperfusion defined as the need for vasopressor agents (norepinephrine, epinephrine, vasopressin, ≥5 μg/kg/minute of dopamine, or ≥50 μg/minute phenylephrine) for greater than or equal to two hours; 3. In patients without known renal disease, renal dysfunction defined as a serum creatinine ≥171 μmol/L or a urine output of less than 500 ml/last 24 hours (or 80 ml/last 4 hours if a 24-hour period of observation not available). In patients with acute on chronic renal failure (pre-dialysis), an absolute increase of ≥80 μmol/L from baseline or pre-admission creatinine or a urine output of <500 ml/last 24 hours (or 80 ml/last 4 hours) is required; 4. A platelet count of ≤50 × 10^9^/L.

Patients were excluded from this trial if they were in the ICU for more than 24 hours prior to enrollment, were moribund, had a contraindication to enteral nutrition, had severe acquired brain injury, had end stage liver disease, had known seizure disorders, were pregnant, or were enrolled in another ICU interventional study. Patients or their next of kin provided informed consent prior to randomization. As per the REDOXS study procedures, patients were randomized to receive glutamine and antioxidant supplementation compared to placebo. Study nutrients continued for 28 days, or until death or discharge from the ICU. Study patients were followed until hospital discharge or death. For surviving patients, contact was made at three and six months to document survival status and health-related quality of life (HRQOL) using the Short Form 36 [8]. The primary outcome for this study was 28-day mortality. The secondary outcomes include duration of stay in ICU, development of infectious complications, multiple organ dysfunction (SOFA scores), duration of mechanical ventilation, hospital length of stay, antibiotic use and costs of care. At baseline, we collected data on patients' admission diagnosis, severity of illness using Acute Physiology and Chronic Health Evaluation (APACHE) II [9] and SOFA scores [3], and the presence of comorbidities using both the Charlson [10] and Functional Comorbidity indices [11].

After 600 patients were enrolled, we performed an interim analysis. Herein, we do not compare across groups as the study is ongoing. Rather, we combined all patients into one dataset to develop and validate POD composite outcome measures. POD is defined as the presence of one or more of: persistent circulatory failure as defined by the ongoing need for vasopressor agents such as norepinephrine, epinephrine, vasopressin, ≥5 μg/kg/minute of dopamine, or ≥50 μg/minute phenylephrine for more than two hours in a given day; persistent renal failure as defined by the need for any ongoing renal replacement therapy; or persistent respiratory/neuromuscular failure as defined by the ongoing need for mechanical ventilation (not including continuous positive airway pressure or non-invasive ventilation) at the outcome assessments time points. A patient was considered liberated from mechanical ventilation if they remained off mechanical ventilation for more than 48 hours. Other organ systems, such as gastrointestinal, neurological, and haematological, were not considered as part of POD because their dysfunction is difficult to quantify reliably in the absence of biochemical tests and does not correlate with the use of specific life-sustaining technologies. For the purposes of this study, we determined the presence or absence of POD at Day 28 amongst survivors. We report prevalence of POD as the proportion of patients with the persistence of organ failure in the individual components of POD and death at Day 28. To validate that patients with POD are different and worse off from those without POD, we evaluated outcomes of patients who survived to Day 28 who had POD and those who did not. We hypothesized that patients with POD would have a higher delta SOFA score, longer duration of ICU stay, longer hospital stay, higher six-month mortality, and lower three and six month HRQOL scores compared to patients without POD and alive at Day 28.

The Research Ethics Board at Queen's University approved the REDOXS study.

### Statistical Analysis

Baseline patient characteristics were compared by POD and survival status at Day 28. Among 28-day survivors, outcomes including Delta SOFA score (maximal SOFA-baseline SOFA), length of stay in ICU, length of stay in hospital, hospital mortality, post 28-day survival and Short Form-36 scores were compared between patients with and without POD at Day 28. Kaplan-Meier curves with log-rank tests are used to compare post 28-day survival between these two groups. ICU and hospital length of stay, defined as days from admission to death or discharge, are described as medians with quartiles and were tested by the Wilcoxon-Mann-Whitney test. Categorical variables are described as counts and percentages and were tested by Fisher's Exact test. All other variables are described as means with standard deviations and compared by the t-test or one-way ANOVA; Welch's test was used if the equality of variance assumption was rejected by Levene's test. All tests are two-sided without adjustment for multiplicity, and a *P*-value < 0.05 was considered statistically significant. Analyses were completed with SAS Version 9.1 (SAS Institution, Cary, NC, USA).

To illuminate the statistical efficiency of POD + death, we compared the sample size requirements based on POD + death to the traditional endpoints of 28-day mortality, ventilator free days (VFD) [12], and organ-failure free days (OFFD) [3] at 28 days. For all these calculations, we use a constant power of 80% and a two-sided alpha = 0.05. Control group event rates and standard deviations were based on the REDOXS study (*n *= 600), but the magnitude of treatment effects were set to arbitrary but typical sizes. The sample size requirements for the time to event endpoints were estimated by the method of Freedman [13], and the other sample size estimates were estimated by *Sample Power Version 2 *(SPSS 2000, SPSS Inc., Chicago, USA) using classic methods [14].

## Results

The first 600 patients enrolled in the REDOXS study were available for analysis for this study. At 28 days, 2.3% of patients had circulatory failure, 13.7% had renal failure, 8.7% had respiratory failure, and 27.2% had died, for an overall prevalence of POD + death = 46.0% (see Table 1). Of 28-day survivors, 20.4% had only one persistent organ failure, 3.0% had two and 2.5% had three. Clinical characteristics of patients with POD, without POD, and who died by Day 28 are shown in Table 2. Patients without POD at Day 28 had lower baseline Charlson Comorbidity scores, APACHE II scores, and SOFA scores compared to survivors with POD (see Table 2).

**Table 1:** Prevalence of the components of POD and death over the first 28 days

	Percentage of patients				
	
ICU day	In shock	On dialysis	Mechanically ventilated	Dead	Dead or with POD*
**1**	84.7	6.2	96.2	0.3	98.0
**2**	81.2	15.0	97.3	2.0	99.7
**3**	55.7	19.0	89.0	5.8	96.3
**4**	35.8	20.7	79.5	8.5	90.8
**5**	24.3	20.2	70.5	10.7	84.7
**6**	17.3	20.5	62.5	12.0	78.8
**7**	14.5	20.5	53.7	13.7	73.8
**8**	13.5	19.8	47.0	15.5	70.0
**9**	13.2	19.3	41.5	16.5	65.3
**10**	11.0	18.8	36.5	17.7	62.7
**11**	9.7	18.5	31.8	18.3	58.5
**12**	8.8	18.7	29.2	19.0	56.8
**13**	6.8	18.3	26.2	20.2	55.0
**14**	6.0	17.5	23.2	21.2	53.0
**15**	5.7	16.5	20.3	22.5	50.7
**16**	6.0	16.0	18.5	23.5	51.0
**17**	5.7	15.7	17.3	24.2	50.7
**18**	4.3	15.0	15.7	25.0	50.0
**19**	3.8	14.5	15.3	25.5	50.2
**20**	4.2	14.2	14.2	25.8	49.7
**21**	3.3	13.8	13.3	26.2	48.8
**22**	3.2	13.8	13.0	26.3	48.8
**23**	2.7	13.8	12.2	26.3	48.0
**24**	2.3	13.8	11.5	26.7	47.8
**25**	1.8	13.8	11.2	26.8	47.8
**26**	1.8	13.8	10.0	26.8	47.0
**27**	2.5	13.7	9.7	26.8	46.7
**28**	2.3	13.7	8.7	27.2	46.0

The proportion of patients with each component of POD, death, and combined POD + death. * This column may be less than the sum of the component columns since patients may simultaneously have more than one POD component.

**Table 2:** Baseline characteristics of patients with and without pod and patients who died by Day 28

	Without POD at Day 28 (*n *= 324)	With POD at Day 28 (*n *= 113)	Dead by Day 28 (*n *= 163)	*P-*value^*a^
**Age**				**<0.001^*bc*^**
	62.6 ± 14.4	61.8 ± 13.8	67.9 ± 13.5	
**Sex**				0.63
Male	198 (61.1%)	70 (61.9%)	93 (57.1%)	
Female	126 (38.9%)	43 (38.1%)	70 (42.9%)	
**Admission type**				**<0.001^*ab*^**
Medical	233 (71.9%)	100 (88.5%)	137 (84.0%)	
Surgical: Elective	42 (13.0%)	6 (5.3%)	8 (4.9%)	
Surgical: Emergency	49 (15.1%)	7 (6.2%)	18 (11.0%)	
**Primary ICU diagnosis**				**0.004^*a*^**
Cardiovascular/vascular	29 (9.0%)	10 (8.8%)	19 (11.7%)	
Respiratory	101 (31.2%)	32 (28.3%)	49 (30.1%)	
Gastrointestinal	5 (1.5%)	2 (1.8%)	6 (3.7%)	
Neurologic	3 (0.9%)	1 (0.9%)	1 (0.6%)	
Sepsis	83 (25.6%)	43 (38.1%)	54 (33.1%)	
Trauma	2 (0.6%)	2 (1.8%)	1 (0.6%)	
Metabolic	7 (2.2%)	1 (0.9%)	4 (2.5%)	
Hematologic	1 (0.3%)	2 (1.8%)	0 (0.0%)	
Other	2 (0.6%)	7 (6.2%)	3 (1.8%)	
Post-op: Vascular/cardiovascular	51 (15.7%)	9 (8.0%)	10 (6.1%)	
Post-op: Respiratory	4 (1.2%)	0 (0.0%)	2 (1.2%)	
Post-op: Gastrointestinal	21 (6.5%)	2 (1.8%)	10 (6.1%)	
Post-op: Trauma	4 (1.2%)	2 (1.8%)	1 (0.6%)	
Post-op: Renal	2 (0.6%)	0 (0.0%)	0 (0.0%)	
Post-op: Orthopedic	1 (0.3%)	0 (0.0%)	2 (1.2%)	
Post-op:Other	8 (2.5%)	0 (0.0%)	1 (0.6%)	
**Charleson Comorbidity Index**				**<0.001 ^*ab*^**
	1.2 ± 1.5	1.7 ± 2.0	1.7 ± 1.7	
**Functional Comorbidity Index**				0.62
	1.3 ± 1.4	1.2 ± 1.4	1.4 ± 1.4	
**APACHE II**				**<0.001^*ab*^**
	24.4 ± 6.6	28.4 ± 5.9	29.1 ± 8.0	
**Day 1 SOFA score**				**<0.001^a*bc*^**
	7.8 ± 2.6	9.4 ± 2.8	8.7 ± 2.9	
**Etiology of shock**				0.16
Cardiogenic	76 (23.5%)	20 (17.7%)	32 (19.6%)	
Septic	203 (62.7%)	81 (71.7%)	112 (68.7%)	
Neurogenic	3 (0.9%)	1 (0.9%)	0 (0.0%)	
Not in shock	7 (2.2%)	4 (3.5%)	2 (1.2%)	
Other	10 (3.1%)	2 (1.8%)	7 (4.3%)	
Hemorrhagic	15 (4.6%)	2 (1.8%)	1 (0.6%)	
Uncertain Origin	9 (2.8%)	3 (2.7%)	9 (5.5%)	

* The global *P*-value tested against the null hypothesis that all three groups have the same mean or proportion is reported. When the global *P*-value < 0.05, unadjusted pairwise comparisons were made. Significant (*P *< 0.05) pairwise comparisons are denoted as follows: a-survivors without POD versus survivors with POD, b-survivors without POD versus decedents, c-survivors with POD versus decedents.

APACHE II, Acute Physiology and Chronic Health Evaluation; ICU, Intensive Care Unit; POD, Persistent Organ Dysfunction Score; SOFA, Sequential Organ Failure Assessment.

**Figure 1:**
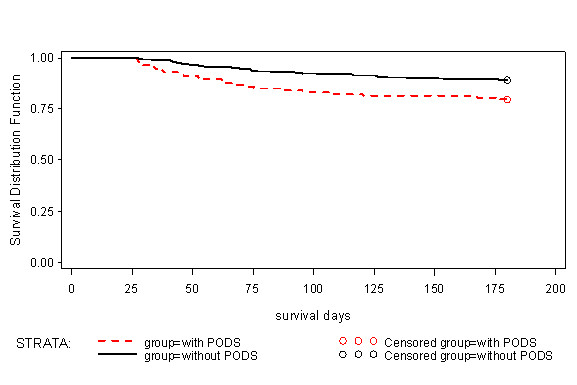
**Kaplan-Meier curve comparing time to death of patients who were alive with and without POD at Day 28**. Patients alive at six months were censored. Differences between curves assessed with log-rank test, *P *= 0.007.

Of survivors at Day 28, those with POD, compared to those without POD, had a significantly longer duration of ICU and hospital stay, and a significantly higher hospital mortality rate and delta SOFA score (see Table 3). In addition, overall mortality from Day 28 to six months was higher in patients alive at Day 28 with POD compared to those without POD (23/113 (20.4%) deaths vs. 35/324 (10.8%), *P *= 0.007, see Figure 1). Finally, patients with POD tended to have a reduced quality of life in many of the domains of the SF-36 at three months (see Table 4). Differences in all domains (except 'General Health Perceptions') were clinically important favoring patients without POD but they only achieved statistical significance for the Physical Functioning (*P *= 0.006) and Role Physical (*P *= 0.005) domains and for the Standardized Physical Component Summary Scale (*P *< 0.001). At six months, there was a trend towards reduced Physical Function scores in patients with POD compared to those without (*P *= 0.08); there were no other differences in any domain scores between the two groups.

**Table 3:** Association of POD with other outcomes in patients surviving to Day 28

	Without POD (*n *= 324)	With POD (*n *= 113)	Total (*n *= 437)	*P*-values
Delta SOFA score (mean ± SD)	2.1 ± 2.2	3.5 ± 3.1	2.5 ± 2.5	**<0.001**
Length of stay in ICU*	8.8 (6.2, 13.4)	28.0 (20.4, 43.9)	10.0 (6.7, 18.3)	**<0.001**
Length of stay in Hospital*	22.1 (13.1, 42.0)	49.0 (30.1, 74.3)	28.3 (15.5, 53.9)	**<0.001**
Hospital Mortality	20 (6.2%)	19 (16.8%)	39 (8.9%)	**0.001**

*Median (first quartile, third quartile) days from admission to discharge or death compared by the Wilcoxon-Mann-Whitney test.

ICU, Intensive Care Unit.

SOFA, Sequential Organ Failure Assessment.

POD, Persistent Organ Dysfunction Score.

**Table 4:** Health related quality of life scores in patients with and without POD at day 28

At 3 months	Patients without POD (*n *= 202)	Patients with POD (*n *= 71)	*P-*value
PHYSICAL FUNCTIONING	50.0 ± 34.1	37.2 ± 31.5	0.006
ROLE-PHYSICAL	43.6 ± 33.0	31.1 ± 27.8	0.005
PAIN INDEX	63.9 ± 30.3	59.0 ± 31.4	0.24
GENERAL HEALTH PERCEPTIONS	53.7 ± 25.3	53.0 ± 21.8	0.84
VITALITY	47.8 ± 25.4	48.2 ± 22.8	0.92
SOCIAL FUNCTIONING	64.9 ± 32.6	62.9 ± 33.0	0.66
ROLE-EMOTIONAL	67.8 ± 31.5	69.3 ± 36.8	0.75
MENTAL HEALTH INDEX	68.9 ± 22.8	72.6 ± 19.8	0.23
STANDARDIZED PHYSICAL COMPONENT SCALE	38.2 ± 12.1	32.7 ± 10.7	<.001
STANDARDIZED MENTAL COMPONENT SCALE	46.4 ± 13.3	49.6 ± 14.1	0.09

**At 6 months**	**Patients without POD (*n *= 196)**	**Patients with POD (*n *= 70)**	***P-*value**

PHYSICAL FUNCTIONING	56.5 ± 34.7	48.1 ± 34.8	0.08
ROLE-PHYSICAL	52.8 ± 34.3	47.4 ± 31.8	0.26
PAIN INDEX	66.3 ± 30.3	65.2 ± 29.4	0.78
GENERAL HEALTH PERCEPTIONS	56.4 ± 26.5	53.3 ± 22.8	0.40
VITALITY	52.5 ± 25.5	53.0 ± 23.8	0.89
SOCIAL FUNCTIONING	71.2 ± 30.3	66.8 ± 33.4	0.31
ROLE-EMOTIONAL	74.1 ± 30.8	70.3 ± 35.0	0.39
MENTAL HEALTH INDEX	71.7 ± 22.4	74.0 ± 21.1	0.45
STANDARDIZED PHYSICAL COMPONENT SCALE	40.4 ± 13.0	38.1 ± 11.4	0.21
STANDARDIZED MENTAL COMPONENT SCALE	48.5 ± 13.3	49.4 ± 13.5	0.63

POD, Persistent Organ Dysfunction Score.

Values reported as mean ± standard deviation.

### Effect on sample size calculations

Table 5 demonstrates the sample sizes needed based on the choice of the primary endpoint for arbitrary but typical effect sizes. At Day 28, 27.2% of the patients were deceased. If we assume a 25% relative risk reduction (RRR) from 27.2% in the control arm to 20.4% in the intervention arm, then we would need 616 patients per arm to achieve 80% power using a Chi-squared test at a two-sided alpha = 0.05. An additional 18.8% of patients had POD at 28 days. Since the rate of POD + mortality is substantially higher than mortality alone, we would require only 286 patients per arm to achieve the same power to detect a 25% RRR from 46.0% to 34.4%.

**Table 5:** Effect of POD+death on sample size estimates

Choice of primary outcome (effect size)	Number required per group
POD + death at 28 days (25% RRR from 46.0%)	286
28-day mortality (25% RRR from 27.2%)	616
VFD at 28-days with SD = 10.2	
(1 day difference)	1,635
(2 day difference)	410
(3 day difference)	183
(4 day difference)	104
(5 day difference)	67
OFFD at 28 days with SD = 11.6	
(1 day difference)	2,114
(2 day difference)	530
(3 day difference)	236
(4 day difference)	133
(5 day difference)	86

Projected sample sizes required to achieve 80% power at a two-sided alpha = 0.05 using control rates and standard deviations observed for the REDOXS Study with arbitrary but typical effect sizes.

OFFD, organ failure free days; POD, Persistent Organ Dysfunction Score; VFD, ventilator-free days.

In the current dataset, the average VFD in 28 days was 12.8 ± 10.2. With this mean and standard deviation, we would need 161 per arm to demonstrate a 25% increase in VFDs. The average of OFFD was 15.3 ± 11.6. We would need a sample size of 146 per arm to demonstrate a 25% increase in OFFDs. Table 5 provides the sample sizes needed for smaller, and more realistic, differences in VFDs and OFFDs.

## Discussion

We have proposed a novel composite outcome measure for use in clinical trials of critical care interventions. We have used a database from a randomized trial of nutrition therapy to demonstrate the prevalence of persistent organ dysfunction amongst survivors of critical illness and to provide realistic estimates for sample size comparisons. We have shown that about a quarter of survivors at Day 28 will still have a need for on-going support from life sustaining technologies in an ICU (POD). These patients who survive to Day 28 and still have POD have a much higher subsequent mortality rate, prolonged hospital course, and reduced quality of life at three months compared to survivors without POD. This is consistent with a prognostic model proposed by Carson and colleagues that showed the patients who undergo prolonged mechanical ventilation (21 days) and have persistent organ dysfunction (need for vasopressor and haemodialysis) have a much greater mortality than those patients with prolonged mechanical ventilation without ongoing organ dysfunction [15].

Composite endpoints are rare in the critical care medicine literature; we are aware of only a few examples. Nathens and colleagues evaluated the effect of an antioxidant supplementation strategy in critically ill trauma patients and reported the combined endpoint of ARDS and pneumonia to reflect overall pulmonary morbidity [16]. In a randomized trial of atrial natriuretic peptide in ischemic acute renal failure in critically ill patients, Sward and colleagues reported on the combined event rate of dialysis or death before or at Day 21 [17]. Schweickert *et al. *evaluated the impact of early physical and occupational therapy on mechanically ventilated patients and chose a primary endpoint of independence with activities of daily living and ability to walk [18]. Finally, Shuster and colleagues conducted a randomized trial of empiric fluconazole in patients at high risk of systemic candidiasis and used a composite that consisted of the following four variables: resolution of fever, absence of invasive fungal infection, no discontinuation because of toxicity, and no need for a non-study, systemic antifungal medication [19]. In no case was a validation exercise, such as we have reported here, conducted to support the use of such composites.

When POD is combined with death to form a composite endpoint, the overall event rate in our dataset increases from 27% for mortality alone to 46% for POD or mortality with increased gains in statistical efficiencies that reduce the sample size required to show clinically important differences compared to when 28-day mortality is used as the primary endpoint. However, the benefits of using POD + death as an outcome measure are more than its impact on statistics and sample sizes. Conceptually, POD + death provide a more comprehensive evaluation of a critical care intervention, evaluating both measures of morbidity combined with mortality. The present reliance on 28-day mortality is not optimal since it would suggest that a patient alive at Day 28 is considered a 'treatment success' even if they are in renal failure and bedridden requiring ongoing mechanical ventilation. To the extent that these surviving patients with major organ dysfunction requiring support occur more frequently in one group in a clinical trial, this important difference will be missed when the focus is on mortality alone and leads to erroneous conclusions about the efficacy of the intervention. However, the validity of a composite measure depends on the extent to which each individual component is similar in its importance to individual patients [20,21]. When the individual components are equally considered as adverse by patients, then it may be appropriate to combine them. However, when there is a gradient of importance across the individual endpoints, it may be invalid to combine them in the form of a composite. For example, investigators evaluated the effect of corticosteroids amongst patients with chronic obstructive pulmonary disease and chose to combine death from any cause, need for mechanical ventilation, and the unblinded administration of corticosteroids together in the form of a composite endpoint [22]. However, from a patient's point of view, it could be argued that a short course of corticosteroids would be judged to be much less of concern than death or the need for mechanical ventilation, which questions the validity of this composite. We have shown that patients surviving at Day 28 with POD will suffer from ongoing risk of serious morbidity, increased mortality, and reduced quality of life at three months. These findings support the argument that POD is similar (but not the same) in magnitude to death and it would be appropriate to combine with death.

Because POD is associated with worse outcomes in the future, some may argue that hospital mortality or longer-term mortality assessments be considered as the primary outcome to judge the efficacy of critical care interventions [2]. We are sympathetic to this notion; however, there are many determinants to mortality unrelated to the ICU illness and the effect of ICU therapy. For example, an 82-year-old patient was admitted to ICU with aspiration pneumonia and respiratory failure following a stroke. Initially, the patient was in shock, requiring vasopressors and had a transient elevation in the serum creatinine. He was enrolled in our REDOXS study and received 14 days of nutrient therapy (or placebo). After two weeks, the patient was liberated from mechanical ventilation and shortly thereafter was discharged from ICU where he had a prolonged stay in hospital convalescing and rehabilitating. Two months after his admission to ICU, he died from complications related to his original stroke and functional disability. This case demonstrates how an ICU therapy may be efficacious in resolving the ICU illness and facilitating a good recovery in the short term but the patient ultimately dies at a time point very remote from his ICU illness. If we use hospital or longer-term time frames to judge the efficacy of our studied ICU therapies, we increase the likelihood that these non-attributable events may occur, resulting in so much noise that it diminishes our effect to detect a signal (that is, the intervention being studied has no chance at altering them) [23]. If we use a time frame more proximate to the ICU admission and application of ICU therapy, we may have greater sensitivity and power to detect treatment effects compared to later outcome assessments. POD + death at 28 days enables a comprehensive assessment of both morbidity and mortality at a time point that will be less likely confounded by random events unrelated to the ICU treatment compared to hospital or longer-term outcomes. Nevertheless, POD + death could be determined at a longer (3 months) or shorter (14 days) time frame depending on the nature of the study. The optimal time to evaluate the outcome determination may vary depending on a number of clinical and methodological factors; and it should be kept in mind that, since POD is time dependent, conclusions may depend on the timeframe selected.

The validity of combining individual events together to form a composite is also dependent on the consistency of the treatment effect across events [20]. For example, we would not want to call a treatment successful if it reduced POD while increasing mortality. However, this is very unlikely since for most patients POD would be on the pathway to death and will be present at the time of death. Composite endpoints may also be misleading if the number of events in the components of greater importance is small compared to the number of events in the components of lesser importance [21]. For example, a statement by investigators that a novel intervention reduces a composite of death, need for dialysis, and reduced serum creatinine is problematic if most of the events were related to a rise in creatinine and investigators found a large apparent treatment effect on rising creatinine but not on the need for dialysis or death. With respect to POD + death, the opposite is found. The most important event, death, occurs much more frequently (27%) than the rates of individual organ failures at 28 days (2 to 14%).

Finally, for composite endpoints to be useful, we need to demonstrate that a critical care intervention has a consistent treatment effect across all individual components before we can have confidence in the interpretation of the composite endpoints. The measurement of a treatment effect can be diluted by combining endpoints, some of which are modified by the intervention, others which are not. For example, investigators studied the effects of carvedilol in patients with left ventricular dysfunction following myocardial infarction [24]. The primary endpoint was the combined rates of all deaths and cardiovascular hospital admission. The study showed that carvedilol was associated with a significant reduction in all-cause mortality (*P *= 0.03) but when combined with hospital admission, there was no overall effect (*P *= 0.30). The mortality effect disappeared when combined with an endpoint that did not change with the intervention. Thus, therapeutic interventions need to have efficacy on all the components before it will be advantageous to combine them. For example, with the mortality and POD rates observed so far in REDOXS, adding POD to a mortality RRR of 25% would only improve efficiency if the RRR of POD was at least 5%. POD and death can 'sensibly' be combined as they are aspects of the same underlying disease process, an underlying inflammatory/immunological process that results in acute organ failure and may result in death or persistent organ failure. We intend to analyze and describe the treatment effects of glutamine and antioxidants on mortality and the individual components of POD with the final results of the REDOXS study when it is completed.

Compared to other options for outcome measures, POD + death offers several other advantages. In contrast to other measures of organ dysfunction/failure which require daily data collection, POD + death requires data collection only on the day of assessment. Furthermore, determination of POD uses readily available clinical parameters that will be easily discerned either prospectively or retrospectively and does not suffer from large amounts of missing data like all other organ failure scoring systems. Compared to traditional mortality endpoints considered in this analysis, POD + death requires fewer study subjects to achieve adequate power to detect a treatment effect. Large differences in 'failure-free days' would require the fewest patients. However, the advantage of POD is its simplicity, lack of missing data, and robustness. To our knowledge, validation exercises similar to what we have performed with POD have not been performed for such endpoints. Finally, by combining death with POD, we avoid the problem of maintaining type I error control over multiple outcomes, and we eliminate the complexities involved with analyzing and interpreting non-fatal outcomes for which death is a competing risk.

The measurement of POD suffers limitations that are common to other organ failure scoring systems. Ideally, scores should be independent of clinician determined therapeutic variables, to minimize bias in the determination of the endpoint [4]. However, this is virtually impossible for all organ failure assessment scoring systems. For example, PaO2/FiO2 ratios will vary dependent on use of positive end expiratory presure, cardiovascular scores will be influenced by use of vasopressors, platelet counts are influenced by platelet transfusions, and so on. For POD, all outcomes assessments are determined by the use of life support technologies, which are determined by clinicians. This limitation will be less important in the context of double-blinded randomized clinical trials.

Other limitations specific to POD is the lack of correlation between POD and six-month quality of life assessment among survivors. This is offset by the presence of POD at 28 days being associated with mortality at six months and may be explained by the fact that patients with a poor outcome have either died or fully recovered by six months.

## Conclusions

Composite endpoints have their limitations. However, if used appropriately, they can improve the statistical efficiency of randomized clinical trials. We propose that POD combined with death may represent a feasible and valid outcome measure for critical care interventions that impact on major morbidity and mortality. POD requires a minimal investment in terms of data collection and provides a comprehensive evaluation of critical care interventions, evaluating both morbidity and mortality. If POD combined with death were used as a primary outcome for relevant critical care studies, more trials for a given investment of research resources could be performed, advancing our knowledge base related to caring for the critically ill. Further validation work to assess the relative magnitude of treatment effects across mortality and the components of POD is warranted.

## Key messages

• Composite endpoints, which combine several clinically related endpoints into an additive outcome measure, are commonly used in other disciplines as a way of enhancing the statistical efficiency and, thereby, reducing the costs of clinical trials.

• Approximately 20% of Day 28 survivors have persistent organ dysfunction (POD) as measured by ongoing requirement for vasopressors, dialysis, or mechanical ventilation at 28 days.

• Of survivors at Day 28, those with POD, compared to those without POD, had a higher mortality rate in the six-month follow-up period, had longer ICU and hospital stays, and a reduced quality of life at three months.

• POD + death may be a valid composite outcome measure and compared to mortality endpoints, may reduce the sample size requirements of clinical trials of critically ill patients.

• Further validation in larger clinical trials is required.

## Abbreviations

APACHE II: Acute Physiology and Chronic Health Evaluation II; HRQOL: health-related quality of life; ICU: Intensive care unit; OFFD: organ-failure free days; POD: Persistent organ dysfunction; RCTs: randomized clinical trials; RRR: Relative risk ratio; SOFA: Sequential Organ Failure Assessment; VFD: ventilator free days.

## Competing interests

The authors declare that they have no competing interests.

## Authors' contributions

DKH was responsible for the overall conduct of the REDOXS trial and conceptualization of the POD analysis. AGD and XJ were responsible for the analysis. All authors contributed to the design and interpretation of this analysis and critically reviewed the final manuscript.
